# Caught on canvas: Facial expressions in art

**DOI:** 10.1177/20416695261468552

**Published:** 2026-08-12

**Authors:** Zoe Thurtle, Daniele Zavagno, Anna Maria Di Betta, Jane Morgan, Olga Daneyko

**Affiliations:** 1 Department of Psychology, Sheffield Institute of Social Sciences, 7314 Sheffield Hallam University, Sheffield, UK; 2 Department of Psychology, University of Milano-Bicocca, Milan, Italy

**Keywords:** emotion, face perception, art, perception

## Abstract

We tested whether facial expressions in artworks can be reliably classified using six basic emotion categories. Participants viewed 116 cropped portraits, selecting the best-fitting emotion for each. Results show that about half of the images elicited consistent responses, aligning with basic emotional prototypes. The rest produced distributed judgments, indicating *Multifaceted Emotions* – layered or uncertain affective signals. The findings support the hypothesis that artists are capable of portraying both basic and complex emotional states, opening up the possibility for a new artwork-based emotion database to study nuanced emotional perception.

## How to cite this article

Thurtle, Z., Zavagno, D., Di Betta, A. M., Morgan, J., Daneyko, O. (2026). Caught on canvas: Facial expressions in art. *i-Perception*, *17*(4), 1–5. https://doi.org/10.1177/20416695261468552

Can facial expressions in artworks be reliably classified into basic emotions? Extensive research has explored facial emotions in photographs and real-life contexts (e.g., Ekman & Friesen, 1971), with the development of important resources based on photography, such as PoFA (Ekman & Friesen, 1993) and ADFES (Van Der Schalk et al., 2011). However, while the six basic emotion model offers a useful framework, dimensional models including valence and arousal may capture subtler variations in the expression of affective states (e.g., Schmidtmann et al., 2020). In such a new framework, visual art may become an interesting tool to study the perception of affective states. While most datasets show static expressions, paintings, though static, often depict dynamic emotional expressions (Actis-Grosso & Zavagno, 2015), possibly portraying affective states beyond the six basic emotions.

Existing studies relating to visual art typically focus on overall mood, not on discrete expressions (e.g., Silvia, 2005), and datasets based on artworks (e.g., Mohammad & Kiritchenko, 2018) provide annotations of emotions evoked by artworks, but they reflect the overall impact on the viewers rather than perceptual judgments of facial expressions. In contrast, the present study isolates facial regions from paintings to examine how facial expressions themselves are interpreted. Although facial regions could theoretically be cropped from existing artwork datasets, such stimuli were not originally designed or validated for facial expression recognition, and therefore lack standardisation in facial visibility, expression clarity, and comparability.

**Figure 1.**
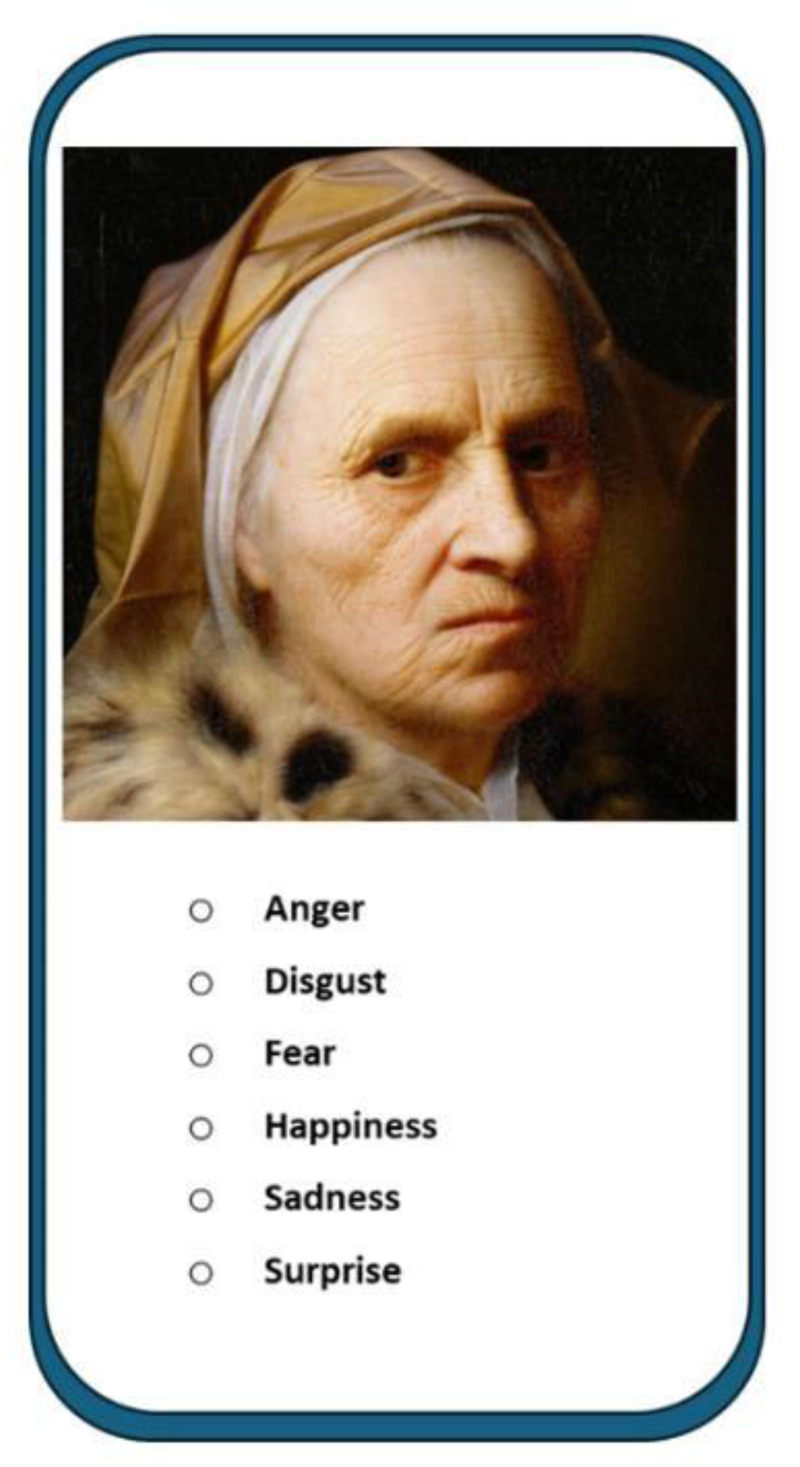
Example of stimulus presentation (Balthasar Denner*, Old Woman*, 1721).

To test the feasibility of a new database of affective facial expressions derived from artworks, we conducted an online experiment via Qualtrics (restricted to mobile phones for consistent presentation), using faces from European paintings (1400–1900), prioritising realistic depictions to minimise stylistic distortion. Selection was guided by facial movements associated with the six basic emotions, informed by FACS (Ekman, 1982), ensuring coverage while preserving variability. We created 116 high-quality standardised images, displayed at ∼6.5 × 6.5 cm. A total of 152 participants (average age = 37 years) were recruited and randomly assigned to one of the two sets. Participants viewed each face individually and selected the most fitting emotion from six basic options (Figure 1). Completion times typically ranged 15–20 minutes.

Each stimulus was analysed independently with Chi-square tests to determine whether emotion distributions deviated from chance. A .05 threshold was adopted, while adjusted standardised residuals (z ≥ 1.96) identified significant trends without Bonferroni correction (Sharpe, 2015). Portraits were classified as Basic Emotion if one emotion accounted for ≥68% of responses and Randolph's free-marginal κ ≥ .41, indicating moderate inter-rater agreement (Randolph, 2005). The 68% agreement threshold was adopted as an empirical decision criterion to identify portraits showing a clear majority consensus among participants and was interpreted alongside κ, which provides an independent measure of agreement beyond chance. All other stimuli were classified as multifaceted.

**Figure 2.**
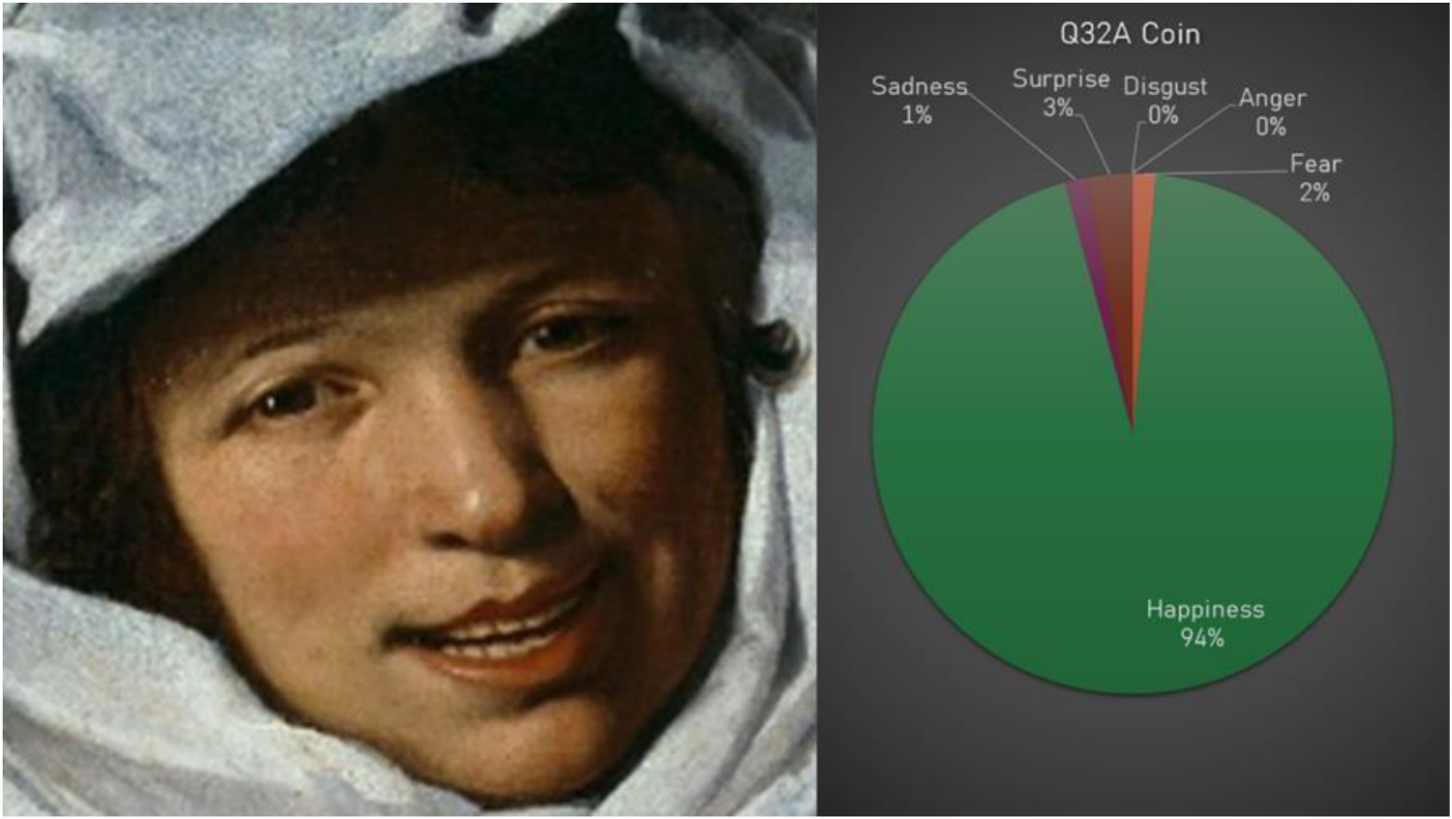
Example of basic emotion: happiness (Bartolomé Esteban Murillo, *The Girl with a Coin*, 1645–1650).

**Figure 3.**
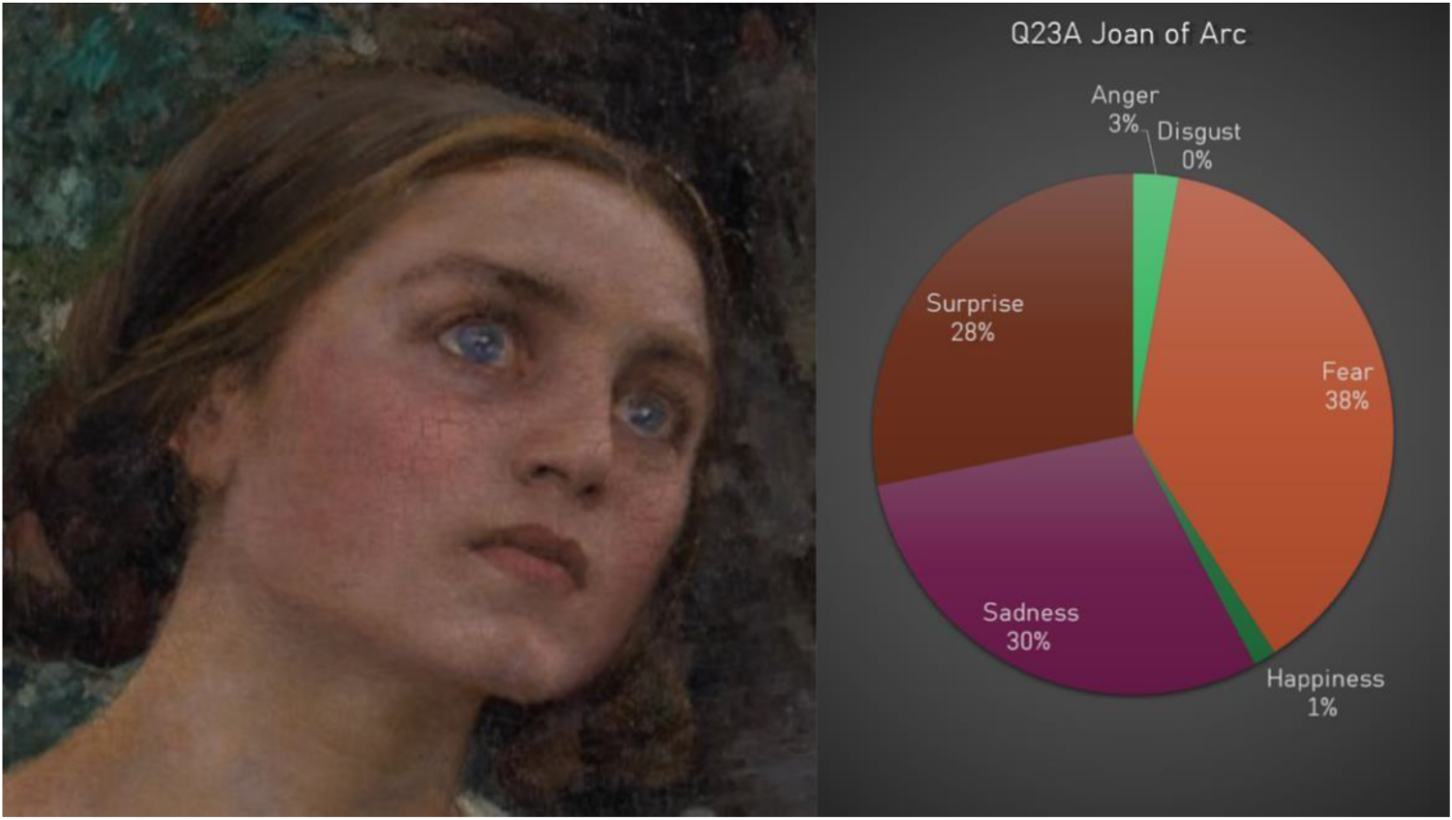
Example of multifaceted emotion (Jules Bastien-Lepage, *Joan of Arc*, 1879).

About 61 stimuli met the criterion to be classified as portraying a Basic Emotion (Figure 2), while the remaining 55 stimuli were classified as Multifaceted Emotion. This suggests overlapping or conflicting emotional cues, sometimes spanning opposing valences. For instance, adjusted standardised residuals highlighted overlapping cues in stimulus 23A – *Joan of Arc*, X^2^ (5, N = 71) = 62.1, p < .001, with fear as the strongest response (z = 4.82), followed by sadness (z = 2.91) and surprise (z = 2.59) (Figure 3). Such results reflect complex, layered affective interpretations.

Results show a balance in our stimuli between canonical prototypes and multifaceted facial expressions, suggesting a continuum of perceptual consensus. Dimensional models (Russell, 2003; Schmidtmann et al., 2020; Takehara & Suzuki, 1997) capture this variation and may guide future work.

Emotion perception depends on partial cues (Carbon, 2020), context (Aviezer et al., 2008), and cultural familiarity (Russell, 1994). Art adds further layers: painters often combine outward expression with allusions to internal states. Renaissance artists, like Leonardo da Vinci, were among the first to recognise the complexity of facial expressions and sought to convey such complexity in paintings. Da Vinci used terms such as *accidenti mentali* (mental states) and *moti mentali* (thoughts) (Zavagno et al., 2022) to speak about subtle psychological states to be portrayed in paintings.

Our study complements recent work on historical trends in painted expressions by Mino and Wijntjes (2026). While they examined temporal and scene-level patterns, we focus on perceptual reliability, highlighting the prevalence of multifaceted emotional signals in art.

The variability observed with these artistic stimuli suggests the opportunity of a new database of facial expressions derived from artworks. The advantage of such a database is that, along with facial expressions classifiable according to the six basic emotions, it would also include expressions representing a broader range of affective states, thus offering an enriched platform for studying emotional perception.
